# Applications of advances in mRNA-based platforms as therapeutics and diagnostics in reproductive technologies

**DOI:** 10.3389/fcell.2023.1198848

**Published:** 2023-05-26

**Authors:** Wjdan S. Bafleh, Haia M. R. Abdulsamad, Sally M. Al-Qaraghuli, Riwa Y. El Khatib, Rawdah Taha Elbahrawi, Azhar Mohamud Abdukadir, Shaima M. Alsawae, Zakia Dimassi, Hamdan Hamdan, Junaid Kashir

**Affiliations:** ^1^ Department of Physiology and Immunology, College of Medicine and Health Sciences, Khalifa University, Abu Dhabi, United Arab Emirates; ^2^ Department of Pediatrics, College of Medicine and Health Sciences, Khalifa University, Abu Dhabi, United Arab Emirates; ^3^ Healthcare Engineering Innovation Center (HEIC), Khalifa University of Science and Technology, Abu Dhabi, United Arab Emirates; ^4^ Department of Biology, College of Arts and Science, Khalifa University, Abu Dhabi, United Arab Emirates; ^5^ Department of Comparative Medicine, King Faisal Specialist Hospital and Research Centre, Riyadh, Saudi Arabia

**Keywords:** oocyte, sperm, embryo, RNA, oocyte activation, calcium, therapeutics

## Abstract

The recent COVID-19 pandemic led to many drastic changes in not only society, law, economics, but also in science and medicine, marking for the first time when drug regulatory authorities cleared for use mRNA-based vaccines in the fight against this outbreak. However, while indeed representing a novel application of such technology in the context of vaccination medicine, introducing RNA into cells to produce resultant molecules (proteins, antibodies, etc.) is not a novel principle. It has been common practice to introduce/inject mRNA into oocytes and embryos to inhibit, induce, and identify several factors in a research context, while such aspects have also been proposed as potential therapeutic and diagnostic applications to combat infertility in humans. Herein, we describe key areas where mRNA-based platforms have thus far represented potential areas of clinical applications, describing the advantages and limitations of such applications. Finally, we also discuss how recent advances in mRNA-based platforms, driven by the recent pandemic, may stand to benefit the treatment of infertility in humans. We also present brief future directions as to how we could utilise recent and current advancements to enhance RNA therapeutics within reproductive biology, specifically with relation to oocyte and embryo delivery.

## Introduction

### mRNA and its therapeutic potential

Messenger RNA (mRNA)-based therapies revolve around the concept of translating exogenous mRNA into functional proteins. Exogenous mRNAs are synthesized by *in vitro* transcription, and a cap analogue is attached to their 5′end for cellular recognition. Since mRNA is largely unstable, targeted delivery requires a form of delivery vehicle which encapsulates the mRNA, such as lipid nanoparticles (LNPs), polyplexes and polymeric nanoparticles, lipopolyplexes (LPPs), and cationic polypeptides. In 1990, the first trial to introduce exogenous mRNA successfully was performed by Wolff et al. (1990), while Martinon et al. (1993) used the concept of transcript mRNA of influenza nucleoprotein to produce a vaccine. The mRNA was encapsulated into liposomes and injected into mice, observing the production of virus-specific cytotoxic T lymphocytes. However, the first clinical application of such an approach was the utilisation of an mRNA-based strategy as a novel rabies vaccine in 2013 (NCT02241135), successfully yielding a functional antibody response targeting viral rabies antigens (Alberer et al., 2017). The most recent example of such technology was also observed in 2020, with the advent of mRNA-based vaccine against Severe Acute Respiratory Syndrome Coronavirus 2 (SARS-CoV-2) (Kim, 2022). Three such vaccines, Pfizer-BioNTech (BNT162b2), Moderna (mRNA-1273), and CureVac101–103 were the fastest vaccines to be developed in medical history, with the Pfizer-BioNTech iteration being the first vaccine to be approved by the FDA for commercialization and use in children 5–11 years old (Yaqinuddin et al., 2021; Fang et al., 2022), with subsequent vaccines also being approved (albeit with some concerns) (Kashir et al., 2022a). Indeed, numerous other applications are also attributed to mRNA platforms within Biomedicine (Table 1).

**TABLE 1:** Summary of applications of mRNA technologies in biomedical applications.

Application	Description	References
Cancer immunotherapy and biomarkers	Utilisation of mRNA encoding for tumor antigens to stimulate the immune system to attack cancer cells. Preclinical/early clinical trials show significant promise for various types of cancer	Kranz et al. (2016), Bareche et al. (2018), Jabulowsky et al. (2018), Burris et al. (2019), Sahin et al. (2020), Sarhadi & Armengol (2022), Shinawi et al. (2022), Sun et al. (2023)
	Large-scale screening studies suggest changes in mRNA expression within tumors could be used in a diagnostic capacity using next-generation screening and microarray approaches, although further detailed investigations are required	
Gene Therapies	Such approaches use mRNA to replace or correct defects in specific genes, with examples including disorders such as cystic fibrosis, muscular dystrophy, diabetes, and cardiac conditions	Carlsson et al. (2018), Gan et al. (2019), Patel et al. (2019), Anttila et al. (2020), Saifullah et al. (2022)
Vaccines for against and infectious diseases	mRNA-produced antigens have the capability of inducing an immune response and contribute towards developing protective immunity against specific viral diseases. The most famous recent example is COVID-19, but also includes diseases ranging from the Zika, Influenza, Cytomegalovirus (CMV), and Rabies viruses	Richner et al. (2017), Stitz et al. (2017), John et al. (2018), Pardi et al. (2018), Sahin et al. (2020), Le et al. (2022), Gote et al. (2023)

The causative factor underlying the global coronavirus disease-19 (COVID-19) pandemic was of course sever acute respiratory syndrome coronavirus-2 (SARS-CoV-2), which enters host cells via the surface S protein - comprising S1 and S2. S1 consists of the receptor-binding domain (RBD), while S2 oversees viral-cell membrane fusion and cellular entry through its cognate receptor - the angiotensin-converting enzyme 2 (ACE2) receptor, which seems to be the case with all current variants of the virus (Kashir et al., 2021; Shafqat et al., 2022). The main antigen target in the case of the COVID vaccine was the S antigen, designed following a 2P mutation, and S1/S2 cleavage site, strategies. In the 2P mutation strategy, two amino acids at the top of the helical position of the S2 subunit center were substituted with prolines (K986P and V987P), to enhance the stability of S protein (Corbett et al., 2020; Wrapp et al., 2020; Zhang et al., 2020). For the S1/S2 cleavage site strategy, part of Q677TNSPRRARYSV687 sequence in wild-type SARS-CoV-2 protein S was deleted to Q677TILRYSV683, causing a change in the amino acid sequence from (RRAR to GGSG), preventing S protein degradation in the host cell. It is cleaved into two subunits, S1 and S2 by the enzyme, Furin, and transmembrane serine protease 2 (TMPRSS2) (Hoffmann et al., 2020a; Hoffmann et al., 2020b). The role of these subunits is to interact with cellular angiotensin-converting enzyme 2 (ACE2), mediating viral fusion to the host cell membrane, resulting in post-fusion confirmation (Fang et al., 2022).

Due to its large molecular weight (104–106 Da), negative charge, and proneness to degradation by nucleases, mRNA cannot pass through the phospholipid bilayer membrane of the host cell. Thus, delivery vesicles such as nano-scale vesicle were developed, composed of structures such as ionizable lipids, helper phospholipids, cholesterol, and polyethylene glycosylated (PEGylated) lipids. These components facilitate the endosomal escape of mRNA, determine the specificity of target organs, and reduce aggregation (Malone et al., 1989; Kauffman et al., 2015; Chen et al., 2016; Cheng & Lee, 2016; Cullis & Hope, 2017; Ball et al., 2018; Sabnis et al., 2018; Hassett et al., 2019; Lokugamage et al., 2019; Buschmann et al., 2021; Fang et al., 2022).

Perhaps most importantly, mRNA vaccine strategies (both ssRNA and dsRNA) exhibit ‘self-adjuvant’ effects, while also inducing antibody production, and adequate immune responses (Alexopoulou et al., 2001; Diebold et al., 2004; Verbeke et al., 2019; Linares-Fernández et al., 2020; Xu et al., 2020). However, regardless of the synthesis/delivery strategy, the concept is that the host cell translates the mRNA sequence by cellular ribosomes to express the antigen of interest, which is degraded into small peptides that will be presented by MHC class I to CD8^+^ cytotoxic T Cells as endogenous antigens. These antigens can be secreted to the extracellular membrane as exogenous antigens and presented by MHC class II to CD4^+^ T Cells (Cagigi & Loré, 2021), which in turn secrete cytokines and activate B Cells for humoral immune response. Upon infection, the immune system recognizes the S antigen on the surface of the virus and triggers humoral and cellular responses (Fang et al., 2022).

### RNA/mRNA processing in oocytes/embryos

RNAs are frequently kept in membrane-free compartments that arise naturally when proteins and/or nucleic acids spontaneously phase separate. Earlier research found many membrane-less compartment types, including P-granules in *Caenorhabditis elegans* (Brangwynne et al., 2009; Seydoux, 2018) and polar granules in *Drosophila* (Malone et al., 1989; Trcek & Lehmann, 2019; Bose et al., 2022), that store mRNAs in non-mammalian oocytes (Flemr et al., 2010; Cheng et al., 2022). Mammalian oocytes actively transcribe a large number of mRNAs. During the last stages of oocyte maturation, transcription arrests until after fertilization when the embryonic genome is activated. The mRNAs that the oocyte/embryo can employ to create new proteins during this time are those that have been stored. Hence, for meiosis to transform oocytes into embryos following fertilization, proper maternal mRNA storage is essential. Cheng et al. (2022) collectively suggested that oocytes of at least mammals, maternal mRNAs, and RNA-binding proteins are primarily deposited around mitochondria.

Interactions between membrane-bound and membrane-less compartments play key roles in cellular architecture and function, according to recent studies. Oocytes undergo a lengthy growth phase during which their mitochondria remain largely inactive. In addition to supplying energy for early embryonic development and oocyte meiotic maturation, maternal mitochondria can produce reactive oxygen species (ROS), which can jeopardize the integrity of the genetic material in the mitochondria and the nucleus (Sasaki et al., 2019; Rodríguez-Nuevo et al., 2022). Fewer ROS are produced, which leads to less DNA oxidative damage (Cheng et al., 2022). This helps to maintain the genetic material’s stability in oocytes. Only fully developed oocytes with surrounded nucleoli (SN oocytes) have completely polarized mitochondria, which provide the necessary energy for oocyte meiotic maturation and subsequent embryogenesis (Cheng et al., 2022).

Jansova et al. (2018) used fluorescent oligo (dT) probes to detect the localization of RNA in the oocyte and two-cell embryos, indicating that whereas the cytoplasm contained comparable amounts of polyA RNA, the embryo’s nucleus contained significantly more than the oocyte’s. To visualize the whole cellular transcriptome, Rolling Circle Amplification (RCA) (Larsson et al., 2010; Lee J. H. et al., 2015) was adopted, and results were similar to RNA FISH; the intensity of fluorescence in the nucleus and cytoplasm was equal in the general transcriptome, while in the 2-celled embryo, the RNA intensity was significantly lower in the cytoplasm. Oocytes store and transcribe mRNA in its growth phase until it reaches its full size, which is approximately 3 mm in cattle, where transcription is decreased. The mRNA’s stability has been proven to depend on the poly(A) tail extension at the 3′ end, whereby the tail’s length is correlated with its capability of successful development; the shorter the poly (A) tail, the lower its competence (Wrenzycki et al., 2007).

Understanding the control of mRNA stability in mammalian oocytes and zygotes has lately made significant advances. In reproductive and developmental biology, the precise processes by which maternal mRNAs are degraded during the maternal-to-zygotic transition (MZT) have long been a matter of debate. The zinc finger protein 36-like 2 (ZFP36L2) protein and CNOT6L, a catalytic component of CCR4-NOT deadenylase, are crucial for mRNA decay that occurs in conjunction with oocyte meiotic maturation. All animal species undergo the MZT, the first stage of early development (Sha et al., 2020), during which maternal gene transcripts are degraded, and the zygotic genome activated. The dynamics of such mRNA and the mechanisms that control the stepwise maternal mRNA clearance during MZT in humans are still unknown, despite such findings in model animals of lower-level species.

Genetic and high-throughput sequencing studies on model systems, including *Drosophila*, zebrafish, and *Xenopus*, have shown that the elimination of maternal transcripts is accomplished by two sequential pathways. The first is entirely mediated by maternal factors accumulated in the mature oocytes and is referred to as maternal (M)-decay; the second depends on *de novo* zygotic transcription products after fertilization and is referred to as zygotic (Z)-decay (Sha et al., 2020). Maternal (M)-decay, entirely mediated by maternal factors accumulated in the mature oocytes, is the first pathway, while zygotic (Z)-decay, which depends on *de novo* zygotic transcription products after fertilization, is the second.

The oocyte-specific adaptor protein of CCR4-NOT, B Cell translocation gene-4 (BTG4), was discovered to be an MZT-licensing factor in mice that mediated mRNA clearance before ZGA9-11 (Morgan et al., 2017; Horvat et al., 2018). Terminal uridine transferase-4/(Tut4/Tut7) and potentially additional early zygotic genes encoding unidentified mRNA destabilizers are transcriptionally activated by the maternal transcriptional coactivator YAP1 and its co-transcription factor TEAD4 (Yagi et al., 2007; Yu et al., 2016; Morgan et al., 2017; Chang et al., 2018). These processes are also important elements of the murine Z-decay system (Sha et al., 2020).

Thus, the RNA landscape in oocytes and embryos is extremely dynamic, and understanding such mechanisms would further allow the development of specific targets using such strategies to be used either therapeutically or diagnostically in the context of specific fertility applications within the clinic.

### mRNA technology for oocyte activation

#### PLCζ/oocyte activation therapeutics

At fertilization, the pivotal signal for oocyte activation in every animal species studied to date is a rise in intracellular calcium concentration (Ca^2+^). In mammalian oocytes, the initial Ca^2+^ rise is followed by repetitive Ca^2+^ transients or ‘oscillations’, triggered by a soluble factor introduced into the ooplasm at gamete fusion by the fertilizing sperm. These oscillations are also observed following intracytoplasmic sperm injection (ICSI; direct injection of a single sperm into the ooplasm) in human and mouse oocytes (Kashir et al., 2010; Kashir et al., 2012a; Nomikos et al., 2017; Kashir et al., 2018). The sperm-specific phospholipase C (PLC), PLCzeta (PLCζ) exhibits the expected properties of the oocyte activating factor (Cox et al., 2002; Saunders et al., 2002), and is considered the strongest candidate for the sperm factor, although several other candidates have also been proposed but without much further evidence or independent support (Parrington et al., 1996; Sette et al., 1997; Sette et al., 1998; Sette et al., 2002; Wu et al., 2007; Kashir et al., 2013b; Aarabi et al., 2014; Nomikos et al., 2015a; Kashir et al., 2015). Interestingly, injection of recombinant mouse and human PLCζ RNA into mouse oocytes not only evoked Ca^2+^ oscillations similar to those induced by sperm, but also promoted subsequent embryo development until the blastocyst stage (Cox et al., 2002; Saunders et al., 2002; Rogers et al., 2004), with a similar response elicited following by microinjection of recombinant mouse PLCζ protein into mouse oocytes (Kouchi et al., 2004; Yoda et al., 2004).

The clinical application of ICSI has been of major importance in human-assisted reproductive medicine in the treatment of male infertility. However, in a small percentage (2%–3%) of such patients, oocytes fail to activate following ICSI and this appears to be due to a deficiency in oocyte activation, specifically due to sperm defects in PLCζ (either reduced/absent levels or abnormal localisation), in even sperm with normal morphology (Yoon et al., 2008; Heytens et al., 2009; Kashir et al., 2014; Saleh et al., 2020). One circumstance where fertilization following ICSI appears to be severely diminished is in globozoospermia, a rare disorder affecting ∼0.1% of infertile men and characterized by round-headed, acrosome-less sperm cells (Liu et al., 1995; Heindryckx et al., 2005; Dam et al., 2006; Fesahat et al., 2019). Such infertile, males also tend to exhibit mutations in the PLCζ gene (Heytens et al., 2009; Kashir et al., 2011b), with numerous such mutations now identified multiple independent studies and correlated with oocyte activation failure in humans (Abdulsamad et al., 2023; Jones et al., 2022; Kashir, 2020). Indeed, PLCζ also seems to be an important indicator of sperm health, correlating with all examined sperm parameters used within the clinic (Kashir et al., 2010; Kashir et al., 2011a; Kashir et al., 2011b; Kashir et al., 2012b; Kashir et al., 2012c; Kashir et al., 2012d; Kashir et al., 2012e; Ramadan et al., 2012; Yelumalai et al., 2012; Kashir et al., 2013a; Nomikos et al., 2013a; Kashir et al., 2013b; Kashir et al., 2014; Yelumalai et al., 2015; Kashir et al., 2016; Kashir, 2020; Kashir et al., 2020; Kashir et al., 2022b; Kashir et al., 2023).

**FIGURE 1:**
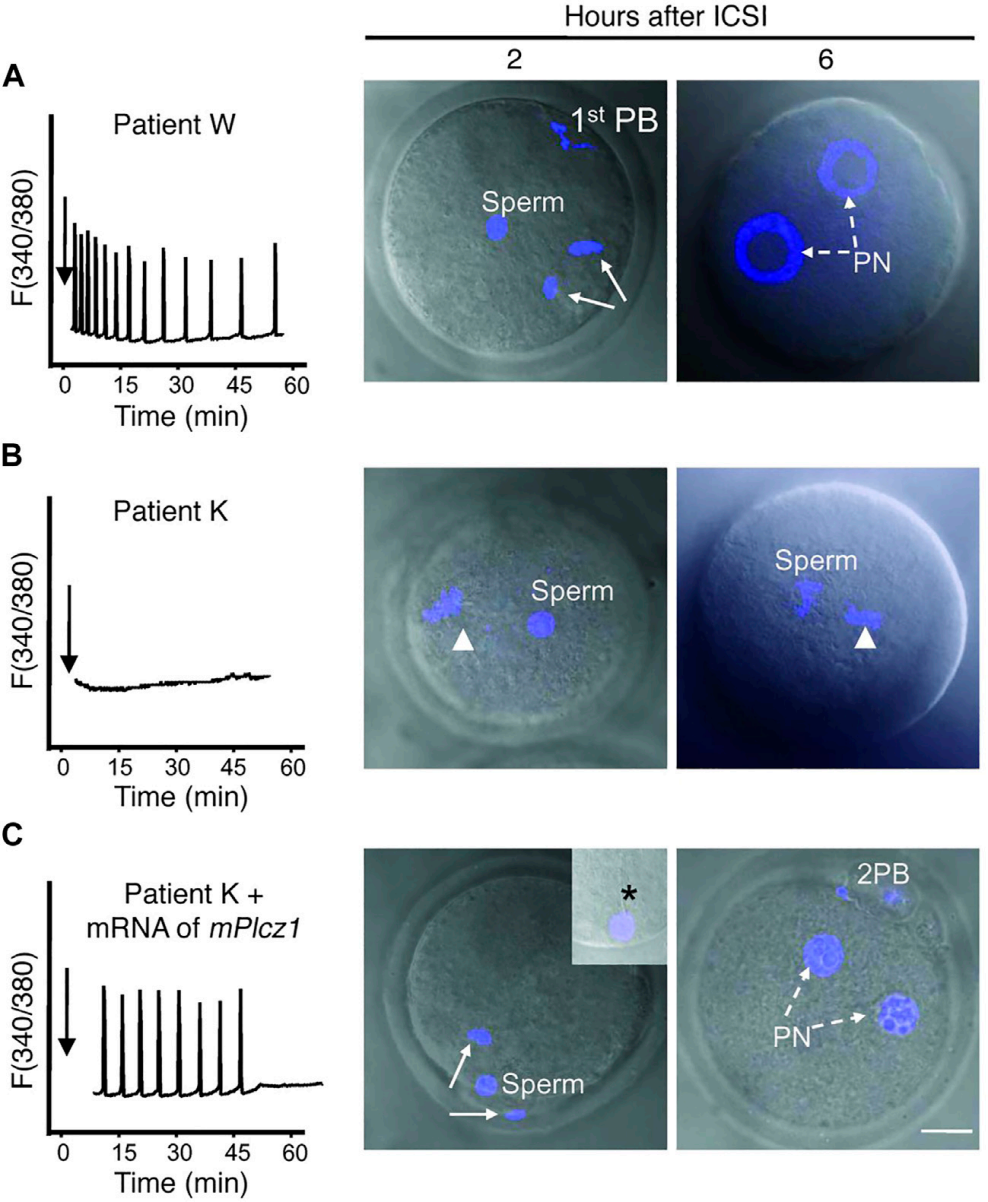
Co-injection of mouse PLCζ mRNA alongside infertile humans sperm in mouse oocytes can rescue Ca^2+^ oscillatory ability. **(A)** Injection of fertile sperm into mouse oocytes exhibited normal Ca^2+^ release patterns and oocyte activation and pronuclear (PN) formation (broken arrows). **(B)** Injection of sperm from an infertile patient (unable to result in fertilisation) was unable to initiate Ca^2+^ release and failed to induce oocyte activation (arrowhead denotes MII chromatin). **(C)** However, co-injection of sperm from the same patient alongside mouse PLCζ mRNA initiated Ca^2+^ oscillations comparable to fertile controls, and enabled resumption of meiosis and PN formation. 1st PB: first polar body; 2 PB: second polar body. Asterisk in inset points to the persistence of the human sperm tail in mouse eggs. Scale bar: 10 μm. Figure adapted from Yoon et al. (2008) with permission.

Most interestingly, co-injection of such sperm with PLCζ mRNA in mouse oocytes was able to ‘rescue’ the induction of Ca^2+^ oscillations, enabling oocyte activation and fertilisation to complete (Yoon et al., 2008; Heytens et al., 2009), the idea of course being that the oocytes active transcriptional machinery converts the RNA to protein, leading to activity. Conversely, injecting mutant, inactive, PLCζ RNA in mouse oocytes failed to elicit suitable Ca^2+^ release, resulting in failed activation in mouse oocytes (reviewed by Kashir, 2020). Collectively, such studies suggest that recombinant PLCζ injection represents perhaps an immensely important therapeutic avenue to rescue cases of ICSI failure (Figure 1). Indeed, this has predominantly been pursued in the form of protein injection (Swann et al., 2012; Nomikos et al., 2013b; Nomikos et al., 2014; Nomikos et al., 2015b; Sanusi et al., 2015), driven predominantly by problems associated with RNA injection into oocytes (discussed later herein). However, this is not to say that injection of such protein is not challenging. Indeed, numerous issues also persist in such applications (reviewed further by Kashir, 2020) leaving some room for improved RNA technology-driven injection of PLCζ.

Another approach could be to target aspects of oocyte activation involved downstream of the action of PLCζ-induced Ca^2+^ release–specifically factors involved in maintaining cell cycle arrest. A major function of oocyte activation (via Ca^2+^ release) is to alleviate arrest through the proteolysis of cyclin B1 by ubiquitin or proteasome activation (Miyazaki and Ito, 2006; Kashir et al., 2022b). Ca^2+^–calmodulin interactions then further repetitively activates calmodulin-dependent kinase II (CaMKII) coincident with each Ca^2+^ peak during fertilization (at least in mouse oocytes), polyubiquitinating cyclin B1 by the anaphase promoting complex/cyclosome (APC/C), a E3 ubiquitin ligase (Swann and Lai, 2016). This entire pathway is inhibited by cytostatic factor (CSF) (Hyslop et al., 2004; Jones, 2004; Jones, 2005; Miyazaki and Ito, 2006), which is inhibited by CaMKII (Hyslop et al., 2004). Finally, Ca^2+^ oscillations also contribute to pronuclear formation by reducing mitogen-associated protein kinase activity (Ducibella et al., 2002; Miyazaki and Ito, 2006).

Ca^2+^ also activates protein kinase C (PKC) to phosphorylate myristoylated alanine-rich C kinase substrate (MARCKS), causing its disassociation from F-actin and leading to actin breakdown in the oocyte cortex, facilitating cortical granule exocytosis. Application of protein synthesis or kinase inhibitors to block the synthesis of cyclin B or CDK1 activity respectively (Heindryckx et al., 2010; Kashir & Swann, 2018), and targeting Emi2 activity (inhibiting cyclin B activity and MPF activity) (Suzuki et al., 2010; Lee K. et al., 2015) are methods that show promise in a supplementary role to Ca^2+^ (Ducibella and Fissore, 2008; Heindryckx et al., 2010; Kashir and Swann, 2018). To this degree, perhaps it would be worthwhile to consider RNA-mediated therapeutic targeting of such factors/processes as an additional/alternative measure to PLCζ activity.

#### Ca^2+^ imaging diagnostics

Ca^2+^ imaging is important in oocytes to confirm Ca^2+^ oscillations and oocyte activation. Multiple tools have been used in the past to image changes in intracellular Ca^2+^, including the photoprotein aequorin in medaka fish, Ca^2+^ sensitive fluorescent dyes such as Fura-2 in sea urchin and hamsters, and calcium green-1-dextran in *Xenopus* (Derrick et al., 2016). The mouse oocyte calcium analysis (MOCA) test quantitatively measures free cytosolic Ca^2+^ spikes in the oocyte using fluorescent probes, and analyzed based on the frequency of Ca^2+^spikes and classified into 4 categories, similar to the mouse oocyte activation test (MOAT), which is used for more severe abnormalities in Ca^2+^ release (Heindryckx et al., 2005; Vanden Meerschaut et al., 2013; Ferrer-Buitrago et al., 2018; Cardona Barberán et al., 2020). On the level of neuronal cells, other kinds of dyes have been used, including Oregon Green BAPTA (OGB)-1 or fluo-4 (Grienberger and Konnerth, 2012; Oh et al., 2019).

However, such methods hold major ethical concerns, with sensitivities of oocytes to Ca^2+^ stimuli perhaps masking specific outcomes Cardona Barberán et al., 2020 Kashir, 2020). If such concerns were to be alleviated, however, there are also other concerns pertaining to Ca^2+^ imaging using fluorescents dyes–namely, that the constant imaging and light exposure exert lethality upon the developing embryo and are thus not used in clinical human IVF and ICSI (Swann et al., 2012). Furthermore, the delivery method of some dyes involves cell permeabilization by a whole-cell patch clamp or with an acute bulk loading protocol that may damage the cell (Oh et al., 2019).

A proposed alternative are genetically encoded calcium indicators (GECIs), which bind to Ca^2+^ ions, based on the Ca^2+^-binding with calmodulin/calmodulin-binding proteins, emitting fluorescence based upon fluorescence resonance energy transfer (FRET), which use a combined form of two fluorescent proteins. Attachment of Ca^2+^ reduces the distance between two proteins to <10 nm, facilitating energy transfer between donor and acceptor, and thus fluorescence emission (Jares-Erijman & Jovin, 2003; Oh et al., 2019). Single-fluorophore based GECI methods are mediated through conformational changes mostly through calmodulin (CaM) and induced chromophore deprotonation leading to elevated fluorescence emission (Nakamura et al., 1999; Akerboom et al., 2009; Oh et al., 2019).

Such methods were have been further modified to examine Ca^2+^ release specifically from acidic organelles, as is the case in oocytes (via the endoplasmic reticulum; ER)—termed genetically encoded Ca^2+^ indicators for optical imaging (GECOs) (Suzuki et al., 2016), which would potentially minimise light-induced damage due to exposure to shorter wavelengths of light, and minimization of reactive oxygen species (ROS) generation (which are generally harmful for cells in large quantities) (Nasr-Esfahani et al., 1990; Squirrell et al., 1999). Satouh et al. (2017) utilized such GECOs, via injection of RNA encoding for various GECOs based upon CaM, to successfully image Ca^2+^ release dynamics without interfering with the efficacy of embryogenesis or birth rates of pups in comparison with controls, whilst also being able to visualize specific Ca^2+^-dependent events such as cortical granule exocytosis. Perhaps such methods would be preferable to using Ca^2+^ dyes, but would of course be subject to limitations associated with RNA use as discussed later. Another aspect to consider is that some GECOs may interfere with Ca^2+^ signalling pathways and it may alter Ca^2+^ oscillations in the oocyte. However, Satouh et al. (2017) and others using similar applications (Cappa et al., 2018; Morita et al., 2021) did not seem to experience such drawbacks.

#### Embryogenic targets

During mammalian oocyte maturation, the female genome is transcriptionally and translationally active, generating a large amount of maternal proteins (De Leon et al., 1983) which are required due to the arrest of transcription prior to ovulation until the 2-cell stage. This MZT process heavily requires large stores of such maternal components to initiate development and activate the embryonic genome (Tadros and Lipshitz, 2009; Li et al., 2013; Zhang et al., 2022). Microarray and proteomic analyses indicate that maternal effect genes affect multiple processes ranging from pronuclear formation, embryogenic cell divisions and gene transcription (Tong et al., 2000; Payer et al., 2003; Tang et al., 2007; Li et al., 2008; Philipps et al., 2008; Kashir et al., 2012b; Li et al., 2013)*.*

**FIGURE 2:**
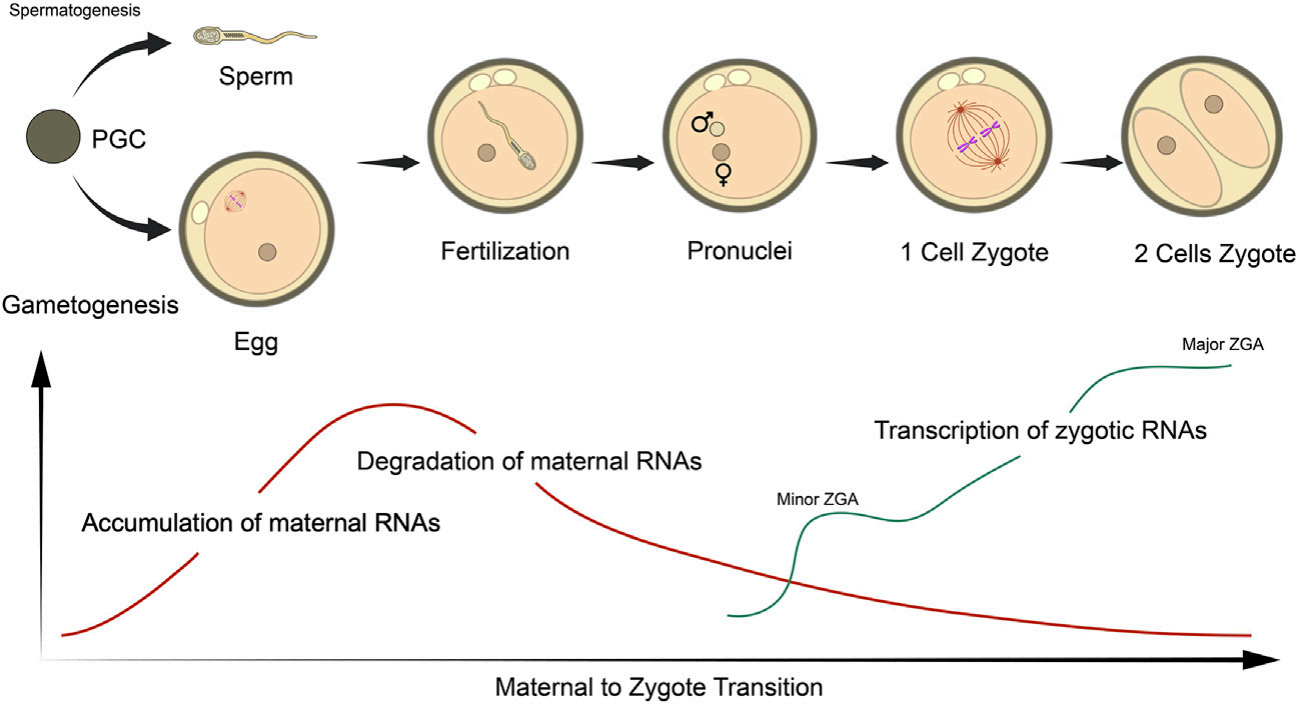
Schematic representation of the maternal to zygotic (MZT) and zygotic genome activation (ZGA) in relation to RNA status in mouse embryos. The MZT initiates following sperm/oocyte fusion at fertilization, which undergoes the various stages of embryogenesis. Oocytes accumulate a large pool of maternal RNA throughout ovulation that are essential for these processes. Post-fertilization, maternal RNAs are gradually degraded, while transcription of embryonic transcripts are initiated at the late zygote stage (minor ZGA) and robustly activated at 2- and 4-cell stages (major ZGA). Figure adapted from Li et al. (2013) with permission.

The initiation of embryonic genome transcription, also known as zygotic genome activation (ZGA) varies among species, ranging from the 1-cell mouse zygote, the 4-8 cell stage in humans and pigs, to the 8–16 cell stage in cows and sheep (Davis, 1985; Braude et al., 1988; Crosby et al., 1988; Frei et al., 1989; Latham et al., 1991; Ram and Schultz, 1993; Bouniol et al., 1995; Li et al., 2013). Large-scale global gene expression profiles seem significantly dynamic, occuring in ‘waves’ during the MZT and preimplantation development in various mammals, during which maternal transcripts are degraded and zygotic genes are activated (Hamatani et al., 2004; Wang et al., 2004; Zeng et al., 2004; Sirard et al., 2005; Whitworth et al., 2005; Zeng and Schultz, 2005; Misirlioglu et al., 2006; Vallée et al., 2008; Li et al., 2013) (Figure 2). For successful embryogenesis, maternal micro RNAs (miRNAs) require degradation concurrent to *de novo* miRNA synthesis at the 2-cell stage (Giraldez et al., 2006; Tang et al., 2007; Bushati et al., 2008; Lund et al., 2009; Ma et al., 2010; Suh et al., 2010), which may be regulated by various short interfering RNA (siRNA) pathways (Tam et al., 2008; Watanabe et al., 2008; Ma et al., 2010).

During the MZT, maternal mRNAs are translated concurrent to specific developmental events that occur before ZGA, dependent upon cis-regulatory elements within transcripts. Such transcripts seem associated with cellular homeostasis and protein biosynthesis (Potireddy et al., 2006; Li et al., 2013). ZGA is also crucial for embryogenesis regulated by multiple regulatory mechanisms involving maternal effect genes, chromatin remodelling and DNA replication (Schultz, 1993; Minami et al., 2007; Li et al., 2013). To this degree, the control of mRNA degradation/translation status is major regulatory step, maintained both spatially and temporally, with specific regulatory factors exerting a critical role during early embryogenesis. Thus, such factors may also serve as important potential targets for RNA-based therapies (knockdown/expression as appropriate) (Teixeira and Lehmann, 2019; Alhajeri et al., 2022; Zhang et al., 2022). Further to maternal factors, Yuan et al. (2023) reported that the paternal genome also exerts a significant influence during maternal RNA degradation (MRD) and ZGA in human early embryos, and mused whether the infertility of some patients may be attributable to defects of paternal contributors of human ZGA.

Some factors that could potentially represent targets for mRNA-based interventions include October 4 (also known as POU5F1), a transcription factor in human embryos, and expressed as maternal transcript and protein in mouse oocytes, linked to cellular pluripotency (Tan et al., 2013; Wu and Schöler, 2014; Cui et al., 2019). Zar1/Zar2 play a critical rule in oocyte meiotic maturation, as well as a major role in inducing 2-cell stage arrest, bind mRNAs, and regulate the stability of the maternal transcriptome and MZT, and trigger mRNA clearance during MZT by interacting with other RNA-binding proteins (Rong et al., 2019). Mater is another essential protein expressed exclusively in oocyte cytoplasms but exert significant effect throughout embryogenesis, remaining present throughout the late blastocyst stages (Tong et al., 2000). Finally, another major player, Nanog, is highly expressed and localized to epiblast, deficiencies in which underlie a failure in appropriate implantation. Importantly, Nanog interacts with numerous other factors (such as CDX2, a Mediator Complex Protein–MED, and Oct4) to regulate status of various RNA transcripts and overall embryonic health (Wu and Schöler, 2014; Cui et al., 2019) (more factors exhaustively reviewed by (Jiang et al., 2023).

All such factors underlying these essential events could represent therapeutic targets for embryos, targeting as appropriate to regulates key events during embryogenesis to perhaps enhance chances of successful pregnancy within the clinic. Another more recent development, however, also would perhaps allow for effective diagnostic analysis of such factors, both in terms of quantification and localisation, within embryos. Jansova et al. (2021) developed a technique further to previous methods (Tanenbaum et al., 2014), using a combination of RNA-FISH and the puromycilation proximity ligation assay, to examine localized translation of non-coding and mRNAs in mouse oocytes and embryos, establishing simultaneous visualization of mRNA and *in situ* translation at the subcellular level, allowing quantitative spatio-temporal analysis. Using such methods, analyses of such RNA also represent significant diagnostic targets.

### Issues associated with RNA-therapies/diagnostics in oocytes and embryos

mRNA therapeutics have given researchers great hope in combating widespread incurable diseases. However, while such technology is associated with certain advantages, such new mRNA technology also exhibit significant drawbacks such as mRNA efficiency, safety, stability, immunogenicity, and enhancement of delivery systems (Table 2; (Almalki et al., 2021; Liu et al., 2021; Pardi et al., 2018; Polack et al., 2020; Sahin et al., 2020; Weng et al., 2020; Zhang et al., 2019). It must be considered, however, that numerous issues pertaining to RNA delivery in the body may not necessarily apply to direct delivery in a much smaller oocyte cell–such as thermodynamic stability. Another example is that while injection of large volumes of RNA solution into the body will result in several hundred thousand–fold dilutions, this issue will not be as profound for oocyte delivery (Guo et al., 2012).

**TABLE 2:** Strengths and weaknesses of mRNA platforms in comparison to current methods.

Strengths	Weaknesses
Specific targeting of factors/disease	Potential for adverse immune response and off-target effects
Rapid development and reconfiguration	Instability and short half-life
No viral or animal requirements	Limited delivery to specific tissues and cells
Sufficient ability to trigger an immune response	Requires specialised transportation and storage
Potential for versatility	May require multiple/repeated dosage to maintain effect
Vast potential for personalised medicine	Limited comparative clinical deployment thus far
Somewhat safer compared to traditional vaccines	

mRNA is a very large unstable molecule, prone to degradation by nucleases (Weng et al., 2020). Indeed, the half-life of mRNA transcribed *in vitro* is a crucial factor hindering application of mRNA-based therapeutics, while natural RNA is extremely sensitive to RNase degradation in the body or within serum (Guo et al., 2012). Rapid progress in recent years have developed chemical base modifications; phosphate linkage modifications; alteration of the 2′carbon (Watts et al., 2008; Singh et al., 2010); use of polycarbamate nucleic acids (Madhuri and Kumar, 2010), locked/bridged nucleic acids (Mathé and Périgaud, 2008); and 5′- and 3′-end capping (Jemielity et al., 2003; Patra and Richert, 2009; Li and Kiledjian, 2010; Ziemniak et al., 2013; Weng et al., 2020), all serving to increase RNase resistance *in vitro* and *in vivo*. However, such modifications may further affect folding and functional properties of the RNA (some more than others) (Hong et al., 2010; Liu et al., 2011; Guo et al., 2012; Weng et al., 2020).

Other chemical modifications include modifying the coding region or the poly(A) tail - plays an important role in regulating the stability and efficiency of the translation of mRNA in union with the 5′cap, the entry site of the internal ribosome, and other factors (Gallie, 1991). Modification of the poly(A) tail could be used to optimize efficiency of mRNA use (Sahin et al., 2014), whereby replacing rare codons with synonymous but frequent codons can improve the translational yield (Gustafsson et al., 2004) via reuse of the same tRNA accelerated translation which occurs due to amino-acylation of tRNAs in the locality of the ribosomes (Cannarozzi et al., 2010). However, a limitation of this method is that each RNA preparation contains a mixture of RNA species that differ in the length of the poly(A) tail (Sahin et al., 2014).

In addition to genetic diseases and cancer, uncontrolled mRNA expression can also result from exposure to environmental toxins or drugs that disrupt normal gene expression patterns. For instance, exposure to heavy metals such as lead can interfere with mRNA processing and transport, leading to an altered mRNA expression pattern. Similarly, certain drugs can affect mRNA synthesis, processing, and stability, leading to unintended consequences for protein production. Another potential drawback suggests that external mRNA introduction could elicit an immunogenic response via Toll-like receptors (Weng et al., 2020). However, such potential issues would perhaps not apply to direct oocyte injection of RNA transcripts.

Most problematic, however, is that data increasingly suggest that mouse zygotes possess a retrotransposon-encoded reverse transcriptase activity, triggered a few hours after fertilization and lasting up to at least the two-cell stage, with products absent in zygotes treated with reverse transcriptase inhibitors (Pittoggi et al., 2003; Sciamanna et al., 2011). This ‘reverse transcription wave’ could generate cDNA products, capable of retention and possibly integration, in zygotic pronuclei and embryonic nuclei. Indeed, it would seem that such activity may even be an essential process driving murine preimplantation development (Sciamanna et al., 2011; Kohlrausch et al., 2022). Indeed, telomere reverse transcriptase (Tert) is highly expressed in oocytes, although this decreased with reproductive age (Blackburn, 2001; Yamada-Fukunaga et al., 2013). Furthermore, expression of long interspersed elements 1 (LINE-1 or L1) - the most common autonomous retrotransposons in humans–is required by mouse embryos alongside activity of endogenous reverse transcriptase for embryogenic development to occur (Beraldi et al., 2006; Kohlrausch et al., 2022).

### Future directions

Rather astoundingly, Ostermeier et al. (2004) revealed that sperm-based mRNAs are transferred to the oocyte. The mRNAs that gain access are degraded, but they play a role in the zygote: for example, clusterin has several roles including, but not limited to, enhancing fertility rate, transport of lipids, and controlling apoptosis. The functions listed, amongst others, are crucial in the early zygote and embryonic development, but not in the oocyte. Yet, as part of the complex paternal contribution, spermatozoa mRNA also supplies vital genomic organelle (the centriole), and male-specific proteomic components (Krawetz, 2005).

**FIGURE 3:**
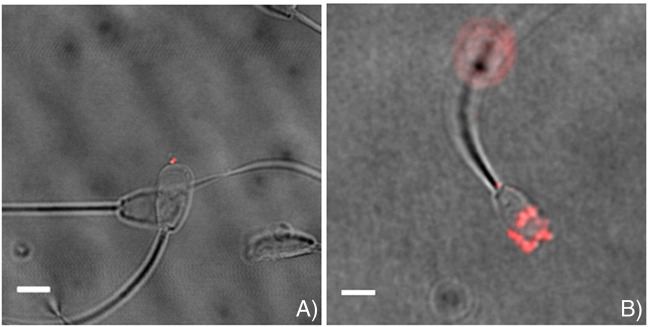
Representative images indicating successful association of loaded mesoporous silica nanoparticles (MSNPs) with sperm. MSNPs were loaded with **(A)** Lamin A/C siRNA, and **(B)** mCherry fluorescent protein. Scalebar = 5 μm. Figure adapted from (Barkalina et al., 2014) with permission.

Considering that sperm is already contributing an RNA-load to the oocyte/embryo, it may be also worth considering using the fertilising sperm as a delivery vehicle to delivery therapeutic/diagnostic RNA. Indeed, Barkalina et al. (2014) showed that spherical mesoporous silica nanoparticles (MSNPs) with hexagonal pore symmetry, loaded common types of cargo (nucleic acids/protein), could form strong associations with porcine sperm following *in vitro* incubation without exerting negative effect upon sperm health (Figure 3), which was preserved following introduction of a cell-penetrating peptide (C105Y) (Barkalina et al., 2015). This was also further applied to mammalian cell (HEK2983T)-derived exosomes, which interacted with boar sperm without affecting parameters of sperm function (Vilanova-Perez et al., 2020) indicating that RNA delivery using such methods could potentially present a less invasive method of introducing RNA to oocytes without injection.

Indeed, numerous other methods also exist to compartmentalize RNA with various encapsulations, including liposome complexes/nanoparticles (Pollard et al., 2013; Pardi et al., 2015; Pardi et al., 2019; Liu et al., 2021; Liu et al., 2021), lipid and other materials-based nanoparticles (Wang et al., 2020; Bahmani et al., 2021; Swetha et al., 2023), and various polymer-based strategies (Zhao et al., 2016; Yang et al., 2023). Another approach worth investigating further is the use of hydrogels, which can be easily modified engineered to deliver RNA in a specific and controlled spatiotemporal manner (Zhong et al., 2023). Such approaches involve a relatively easy mechanism of loading for naked/single stranded RNA (Zhong et al., 2023), and may perhaps minimise potential reverse transcriptase activity. However, the efficacy of such a system needs further evaluation in the context of gametes/embryos.

## Conclusion

The use of mRNA therapeutics in the context of specific diseases has matured rapidly in recent years, accelerated further by the devastating COVID pandemic, maturing into a range of improved modifications and delivery systems that have proven to be extremely effective in generating effective responses against specific viral infections. Such strategies present significant promise for other non-viral infectious diseases, as well as numerous types of cancers.

**FIGURE 4:**
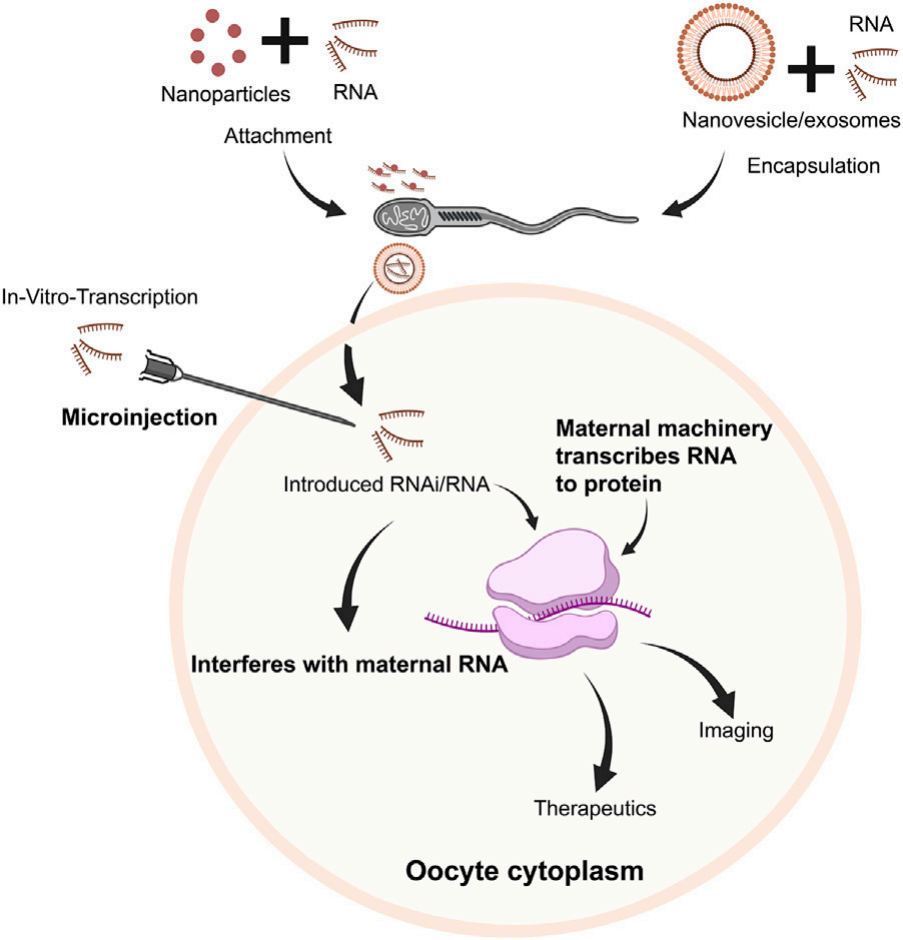
Schematic overview of potential pathways of RNA use within oocytes/embryos. Following *in vitro* transcription, RNA transcripts (mRNA/RNAi, etc.) could be injected into oocytes and embryos. Alternatively, RNA transcripts could either be attached with nanoparticles, or encapsulated in nanovesicles/exosomes and attached/associated with sperm, which could be delivered to the oocyte along with RNA cargo. RNAi could downregulate specific pathways to regulate embryogenesis, targeting specific genes/transcripts. Concurrently, mRNA would be translated to protein, serving therapeutic or diagnostic purposes.

Herein, we discuss another potential area wherein mRNA therapeutics could represent a significantly powerful tool to perhaps enhance quality and success of fertility treatments *in vitro*–specifically within the context of delivery and expression within oocytes and embryos (Figure 4). Indeed, RNA has been traditionally a powerful tool in an extensive repertoire of methods used to study reproductive biology. However, such applications have been traditionally bereft with limitations and safety considerations, which have prevented their application in a clinical format both as a therapeutic and potential diagnostic. As we discuss, however, given the recent advances and improvements in mRNA delivery, stability, and efficiency, perhaps it is time to reconsider.
